# A multimodal study and management of retinitis punctata albescens

**Published:** 2020

**Authors:** Glenda Espinosa-Barberi, José Francisco Galván González, David Viera Peláez

**Affiliations:** * Institut Català de Retina, Barcelona, Spain; ** Postgraduate and Doctorate School, University of Las Palmas de Gran Canaria, Las Palmas de Gran Canaria, Spain; *** Hospital Universitario de Gran Canaria Doctor Negrín, Ophthalmology Department, Las Palmas de Gran Canaria, Spain

**Keywords:** retinitis punctata albescens, electroretinogram, visual field, optical coherence tomography

## Abstract

**Purpose:** To study disease progression and visual function in a patient with retinitis punctata albescens (RPA).

**Method:** Observational case report. The retinaldehyde-binding protein 1 gene (RLBP1) was analyzed by direct genomic sequencing. A complete ophthalmologic examination was performed.

**Results:** Mutations in the RLBP1 gene were identified in the patient. The patient’s fundus (OF) showed numerous white dots with diffuse retinal mottling. Her visual function deteriorated progressively during the follow-up. Optical coherence tomography (OCT) demonstrated bilateral cystic macular edema that worsened if the patient stopped dorzolamide topical therapy.

**Conclusions:** The multimodal study is useful in the characterization of retinal dystrophies, in association with neurophysiological tests. Degenerative changes of the outer retina were detected by OCT.

**Abbreviations:** RPA = Retinitis punctata albescens, RP = retinitis pigmentosa, IOP = Intraocular Pressure, BCVA = Best Corrected Visual Acuity, OD = right eye, OS = left eye, OU = both eyes, BMC = biomicroscopy, AF = autofluorescence, OF = ocular fundus, ERG = electroretinogram, OCT = optical coherence tomography, VF = visual field, VEP = visual evoked potentials, CME = cystic macular edema, MD = mean deviation, RLBP1 = retinaldehyde-binding protein 1

## Introduction

Retinitis punctata albescens (RPA) is a progressive retinal rod-cone dystrophy, primarily of autosomal recessive inheritance, considered an atypical or incomplete variant of retinitis pigmentosa (RP) [**1**]. It is characterized by the funduscopic finding of rounded white-yellowish deposits in the retina, especially at the level of the equator. It is associated with nyctalopia, decreased visual acuity (BCVA), campimetric alterations, and reduction or absence of response to stimuli in the electroretinogram (ERG), as well as RP [**2**,**3**].

## Case report

**Fig. 1:**
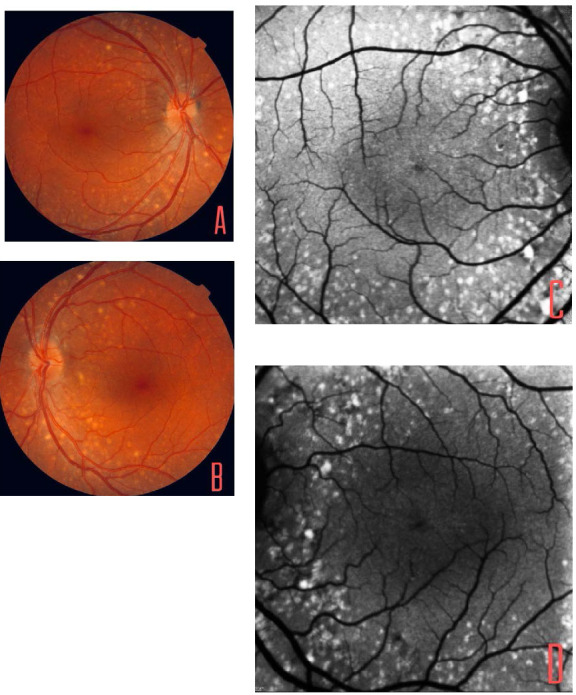
**A,B** Color retinographies: Presence of numerous white-yellowish rounded lesions of subretinal appearance that respect the macular area. **C,D** Autofluorescence: Hyperreflectivity corresponding to subretinal lesions

We presented a 10-year-old woman who had nyctalopia as the main symptomatology. She presented no personal or family history of interest, except hyperopia of +9 diopters. In the ophthalmological examination, the BCVA was 20/ 20 in the right eye (OD) and 20/ 25 in the left eye (OS). Biomiscroscopy (BMC) and intraocular pressure (IOP) were normal. In OF, yellowish rounded subretinal lesions were observed in both eyes (OU), following the distribution of the temporal vascular arches, respecting both macules (**Fig. 1 A,B**). The OCT did not show alterations at the macular level. In autofluorescence (AF), hyperfluorescent lesions corresponding to subretinal lesions were observed, without other pathological findings (**Fig. 1 C,D**).

Due to these findings, complementary neurophysiological tests such as visual evoked potentials (VEP) and electroretinogram (ERG), and visual field (VF) 30-2, were requested. The VEPs showed a regular morphology with wave latency peaks and amplitudes within normal limits, while the ERG revealed some irregular morphology potentials, collecting the latencies of a waves and conserved and increased the b waves in OU, both in photopic and in scotopic conditions.

The VF 30-2 showed peripheral arciform defects and general reduction of sensitivity in OU, with a mean deviation (MD) of -5.93 dB (p <1%) in OD and -7.46 dB (p <0,5%) in OS, and with a standard deviation above the average of 1.91 dB in OD and 1.82 dB in OS. The genetic test was positive for the mutation in the gene that codes for retinaldehyde-binding protein 1 (RLBP1), so the diagnosis of retinitis punctata albescens was confirmed.

**Fig. 2:**
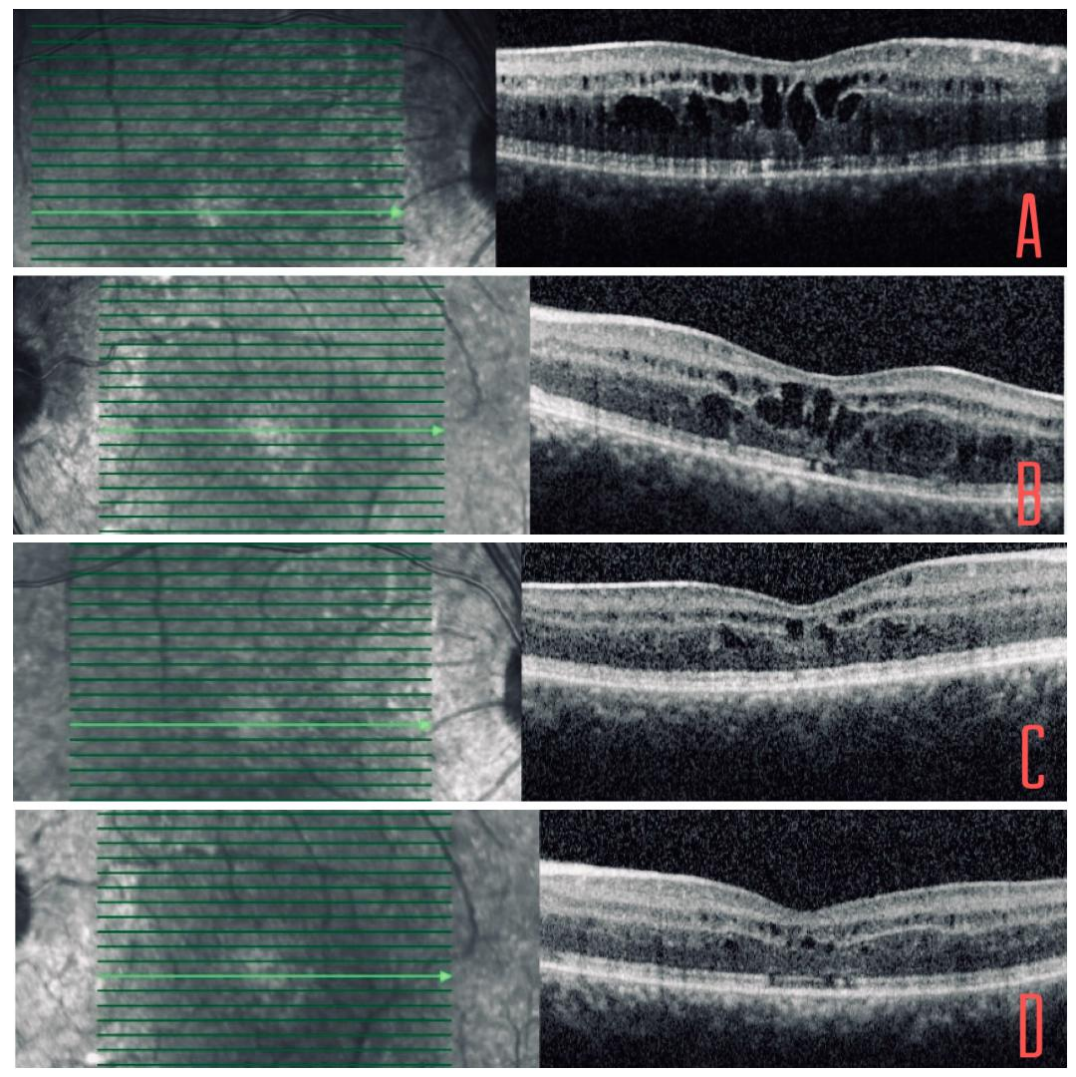
**A,B** Macular OCT at the onset of cystic macular edema in which foveal profile involvement and irregularities in external layers can be seen. **C,D** Current macular OCT with control of cystic macular edema due to chronic treatment with topical dorzolamide

In subsequent reviews, the patient presented several episodes of cystic macular edema (CME) of less than 500µ, which have been partially controlled by treatment with topical dorzolamide every 12 hours, occasionally using different oral acetazolamide guidelines. Due to the worsening of the CME after the suspension of the topical treatment, the application of topical dorzolamide has been maintained chronically. In the last examination, the BCVA was 20/ 30 in OU, the lesions described in the OF did not present changes and small intraretinal cysts could be seen in the macular OCT (**Fig. 2**).

## Discussion

RPA is a rare form of RP. This rod-cone dystrophy has an incidence of 1/ 800,000 cases worldwide [**3**].

Various forms of transmission have been described, the autosomal recessive inheritance being the best known, generally due to mutations in the RLPB1 gene, although other associated genes have been described, as well as mutations in this gene, relations with other retinal dystrophies, such as Bothnia retinal dystrophy (BD), Newfoundland rod-cone dystrophy (NFRCD), and fundus albipunctatus (FA), so it can be stated that there is heterogeneity of phenotypic expression, according to the structure of the compromised protein [**4**-**6**].

The RLPB1 gene codes for the so-called cellular retinaldehyde-binding protein 1 (CRALBP), whose function is to bind to 11-cis-retinol that will be transformed into 11-cis-retinal to form visual pigments in both cones and rods, have as main manifestation the reduction of vision, starting with nyctalopia [**7**,**8**].

As observed in the case described, the same clinical manifestations as in a pigmentary retinopathy are present in the disease, with the OF in salt and pepper and characteristically the appearance of well-defined and rounded white-yellowish lesions, whose origin is at the level of the pigmentary epithelium [**2**,**9**].

Because the function of the photoreceptors is affected by the pathophysiological process, neurophysiological tests confirm the deterioration and, in advanced stages, the abolition of the responses of both cones and rods may be present [**1**].

The visual prognosis depends on the degree of macular atrophy generated during the course of the pathology, which is usually progressive [**2**].

## Conclusion

In conclusion, the case presented is a rare association between the RPA and the CME, in which, besides the dystrophy itself, we faced the challenge of the management of persistent CME within the pathology, probably caused by the incompetence of the RPE, which worsened the patient’s visual prognosis.

Formerly, the diagnosis was based on clinical and neurophysiological tests. At present, a more complete study can be carried out to characterize the damage of the retinal outer layers, as well as the possible additional complications.

**Acknowledgments**

None.

**Sources of funding**

Authors have not received founding from any organization related (National Institutes of Health (NIH); Wellcome Trust; Howard Hughes Medical Institute (HHMI).

**Disclosures**

The authors declare that they have no links of interest in relation to this article.
